# Estimating disability-adjusted life years for breast cancer and the impact of screening in female populations in China, 2015–2030: an exploratory prevalence-based analysis applying local weights

**DOI:** 10.1186/s12963-022-00296-1

**Published:** 2022-10-07

**Authors:** Xin-Xin Yan, Juan Zhu, Yan-Jie Li, Meng-Di Cao, Xin Wang, Hong Wang, Cheng-Cheng Liu, Jing Wang, Yang Li, Ju-Fang Shi

**Affiliations:** 1 Office of Cancer Screening, National Cancer Center/National Clinical Research Center for Cancer/Cancer Hospital, Chinese Academy of Medical Sciences and Peking Union Medical College, 17 Panjiayuan South Lane, Chaoyang District, Beijing, 100021 China; 2 National Central Cancer Registry, National Cancer Center/National Clinical Research Center for Cancer/Cancer Hospital, Chinese Academy of Medical Sciences and Peking Union Medical College, Beijing, 100021 China; 3 Department of Statistics and Information, Beijing Center for Disease Prevention and Control, Beijing, 100013 China; 4 Division of Health Information Dissemination, Institute of Medical Information, Chinese Academy of Medical Sciences and Peking Union Medical College, No. 3 Yabao Road, Chaoyang District, Beijing, 100020 China

**Keywords:** Breast cancer, DALYs, Screening, Disability weights, China

## Abstract

**Background:**

Most cancer disability-adjusted life year (DALY) studies worldwide have used broad, generic disability weights (DWs); however, differences exist among populations and types of cancers. Using breast cancer as example, this study aimed to estimate the population-level DALYs in females in China and the impact of screening as well as applying local DWs.

**Methods:**

Using multisource data, a prevalence-based model was constructed. (1) Overall years lived with disability (YLDs) were estimated by using numbers of prevalence cases, stage-specific proportions, and local DWs for breast cancer. Numbers of females and new breast cancer cases as well as local survival rates were used to calculate the number of prevalence cases. (2) Years of life lost (YLLs) were estimated using breast cancer mortality rates, female numbers and standard life expectancies. (3) The prevalence of and mortality due to breast cancer and associated DALYs from 2020 to 2030 were predicted using Joinpoint regression. (4) Assumptions considered for screening predictions included expanding coverage, reducing mortality due to breast cancer and improving early-stage proportion for breast cancer.

**Results:**

In Chinese females, the estimated number of breast cancer DALYs was 2251.5 thousand (of 17.3% were YLDs) in 2015, which is predicted to increase by 26.7% (60.3% among those aged ≥ 65 years) in 2030 (2852.8 thousand) if the screening coverage (25.7%) stays unchanged. However, if the coverage can be achieved to 40.7% in 2030 (deduced from the “Healthy China Initiative”), DALYs would decrease by 1.5% among the screened age groups. Sensitivity analyses found that using local DWs would change the base-case values by ~ 10%.

**Conclusion:**

Estimates of DALYs due to breast cancer in China were lower (with a higher proportion of YLDs) than Global Burden of Disease Study numbers (2527.0 thousand, 8.2% were YLDs), suggesting the importance of the application of population-specific DWs. If the screening coverage remains unchanged, breast cancer-caused DALYs would continue to increase, especially among elderly individuals.

**Supplementary Information:**

The online version contains supplementary material available at 10.1186/s12963-022-00296-1.

## Introduction

Increasing attention has been given to disability-adjusted life years (DALYs) [1]. DALYs have been regarded as a common indicator of burdens of disease in many countries [2]; DALYs consider both the burden of death (years of life lost [YLLs] due to premature death) and the years lived with disability (YLDs). Population-specific disability weights (DWs) play an important role in the accuracy of YLDs estimations [3, 4]. The Global Burden of Disease Study (GBD), an influential global study, evaluated the DALYs associated with more than 300 diseases and injuries. Early DWs of GBD were obtained from five countries (such as Bangladesh), and since 2013, data from four European countries have been added [5]. However, it can still not distinguish the DWs and YLDs with high precision in the context of different social and cultural backgrounds [3, 4]. Population-specific breast cancer DWs are of great value in DALYs estimations at the population level in China and worldwide.

In 2015, 304 thousand new cases of female breast cancer occurred in China [6], ranking first among female cancers. With the aging of the population, the burden of breast cancer would keep an upward trend [7, 8]. The 5-year survival rate among female breast cancer patients in China was 82.0% between 2012 and 2015. Compared with that of advanced-stage breast cancer, the prognosis of early-stage breast cancer is better [9], and the cost of diagnosis and treatment was lower [10, 11]. Breast cancer screening is becoming an essential strategy for cancer prevention and control. A national screening program for breast cancer in Australia has observed a decline in the burden of breast cancer [3]. A recent systematic review including 27 global studies showed a 22% reduction in breast cancer mortality after carrying out breast cancer screening for approximately a decade [12]. Females have benefited greatly from several national screening programs. The central government has proposed the “Healthy China initiative (2019–2030)” that the coverages of breast cancer screening in rural areas are expected to reach 80% and 90% by 2022 and 2030, respectively [13]. Overall, the impact of breast cancer screening should be considered in predictions of burden of breast cancer in the long term.

A large-scale study on quality of life in China, covering 12 provinces and covering nearly 100 thousand people, reported the population-specific health utility of breast cancer [14–16] and the team also evaluated and compared the disability weighting methods systematically [17–19], which provide potential access for the evaluation of population-specific DWs with high precision. Most previous cancer DALY studies worldwide used broad, generic DWs; however, differences exist among populations and cancer types. Therefore, using breast cancer as an example, this study aimed to estimate population-level DALYs among Chinese females and the impact of applying local and detailed DWs.

## Materials and methods

### Overall design

**Fig.1:**
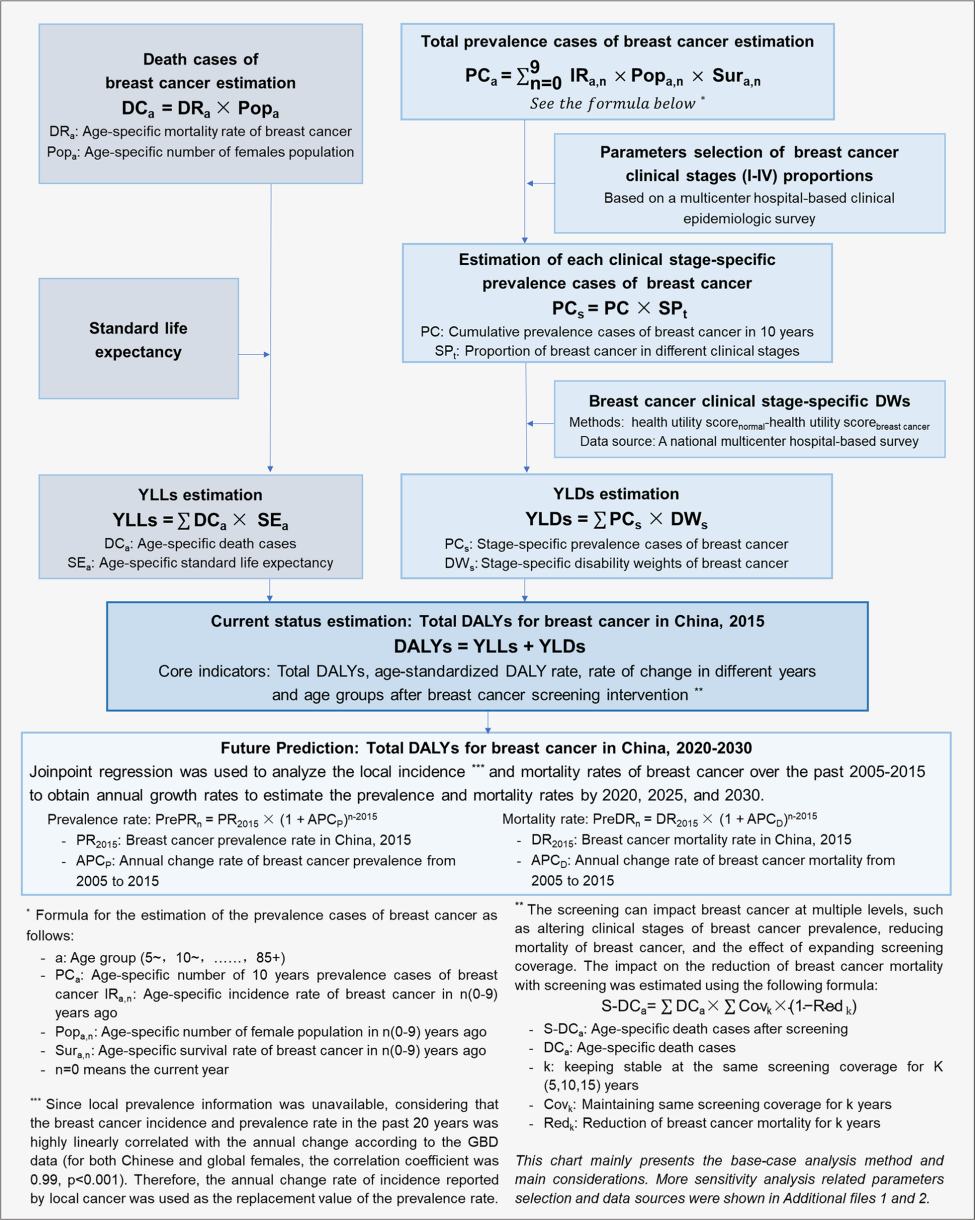
The overall methodology

Using available multisource data (including published literature, open databases and local studies), a prevalence-based model was built [5]. Then, Joinpoint regression was applied to predict the prevalence and mortality rates of breast cancer and associated DALYs from 2020 to 2030. Finally, sensitivity analyses were performed to evaluate the impacts of demographics, DWs and screening parameters. The analysis indicators were DALYs and the age-standardized DALY rate (ASDR) considering Segis’ world standardized population. The overall methodology was shown in Fig. 1.

### Data sources

Data on the incidence, mortality, and DALYs rates of breast cancer in China from 2006 to 2015 were extracted from the Chinese cancer registry annual reports (2009–2018) [20–29]. A national cancer surveillance network exists in China, and cancer registration is carried out in all provinces. In 2015, the national cancer surveillance network was expanded to include 501 cancer registries and covered 387,872,825 people, including 197,211,672 males and 190,661,153 females, accounting for 28.22% of the national population. All patients included in the registry had invasive cases, and all cancer cases were coded according to the International Classification of Diseases for Oncology, 3rd revision (ICD-O-3), and the International Statistical Classification of Diseases and Related Health Problems 10th Revision (ICD-10). Provincial cancer registry centers are responsible for collecting, evaluating, and publishing provincial cancer statistics (including incidence, mortality and survival). All hospitals, medical and health institutions in administrative regions are required to submit cancer records to local cancer registries; this data is then submitted to the national cancer registry [29].

Demographic data were obtained from officially published reports, including China Population Statistics Yearbook [30] and World Population Prospects 2019 [31], which contain age-specific demographic information. Female standardized life expectancies were obtained from the GBD 2015 [32] and World Population Prospects 2019 [31]. Epidemiological parameters of breast cancer (incidence rate, mortality rate and survival rate) were obtained from the Chinese Cancer Register annual report [20–29] and the previous study [9]. DWs were obtained by converting the health utility values (calculated by “health utility score_normal_-health utility score_breast cancer_”) [18, 19], with the data of utilities obtained from two Chinese studies [15, 18, 19, 33]. This method was the most commonly used method summarized by a previous systematic review (five calculation measures in total) [17]. The proportions of clinical stages were obtained from national research in China [34, 35]. The detailed parameter sources and methods were shown in Additional files 1 and 2.

### Parameters of breast cancer screening

Previous studies have shown that breast cancer screening plays a vital role in disease progression and the distribution of disease factors, such as the distributions of clinical stage and mortality. (1) The distributions of clinical stages were calculated from base-case data obtained from a multicenter hospital-based clinical epidemiologic survey among females who did not undergo screening in 2015; the population included 4211 breast cancer patients from 7 provinces in China (stage I: 19.2%) [35]. The data of Chinese females who underwent screening in 2015 were obtained from the “Central Financial Transfer Payment Project-Chinese Women Breast Cancer Screening Study”, which included 0.4 million women from 30 provinces in China (stage I: 35.9%) [34]; (2) The ten-year reduction in breast cancer mortality after screening was calculated from base-case data from a recent global meta-analysis (including 27 studies), which reported that the integrated mortality reduction rate was 22% (95% confidence interval [CI]: 18–25%)[12]. A linear hypothesis that the breast cancer mortality after screening five years and 15 years declined by 11% and 33%, respectively, was made. According to the results of the global meta-analysis and hypothesized data, “42%” was the maximum value of the included studies in the meta-analysis; since there were differences among the included studies, the sensitivity analyses tried the values of 25% (upper 95% CI of the meta-analysis results), 15%, and 5% in turn. (3) The coverage of breast cancer screening: According to the population-level screening coverage rates reported in the previous studies in China, breast cancer screening rates were 25.7% in women aged 35–64 years in 2015, and increased approximately 1% per year from 2013 to 2015 [36, 37]. Accordingly, the coverage rates (at the individual level) of breast cancer screening in Chinese females were estimated to be 30.7%, 35.7%, and 40.7% for 2020, 2025, and 2030, respectively.

### YLDs estimation

The incidence rates of breast cancer from 2006 to 2015 in China were derived from the annual report of Chinese cancer register[20–29] and female population statistics[30], using numbers of females and new breast cancer cases, and local survival rates, to obtain the overall numbers of prevalence cases in 2015 according to the prevalence calculation formula[38] (see Fig. 1 Formula 1). Then using local data(detailed year- and age-specific data were shown in Additional file 3) on clinical stage-specific(I-IV) proportions for breast cancer and DWs to calculate overall YLDs. Survival rates were based on the 5-year survival rate of breast cancer in 2012–2015 in China (the best available local data at present) [9] and the survival rate ratios between age groups calculated from Surveillance, Epidemiology, and End Results (SEER) 5-year relative survival rates [39]. We extrapolated the age-specific 1- to 9-year survival parameters of breast cancer in China (detailed year- and age-specific data were shown in Additional file 3). The base-case DWs for different clinical stages (I–IV) were calculated as “health utility score_normal_-health utility score_breast cancer_”, which was the most commonly used method in previous systematic review [17–19]. The health utility scores were extracted from two previous Chinese articles [15, 34].

### YLLs estimation

Total YLLs were estimated using mortality rates of breast cancer, female numbers and standard life expectancies [29, 30, 32]. The standard life expectancy (84.2 years) estimated by the GBD 2015 was used as the base-case parameter for external comparison [32]. Detailed year- and age-specific life expectancy data were shown in Additional file 4. According to the standard life expectancy in 2015 reported by the GBD 2015 and in 2050 reported by the World Health Organization (WHO) [40], the standard life expectancy in 2030 was estimated linearly, detailed age-specific data were shown in Additional file 4.

### Predicting DALYs for 2020–2030

The incidence (strong linear correlation with the annual change rate of prevalence, correlation coefficient = 0.9, *p* < 0.001) and mortality rates of breast cancer were derived from the annual reports of Chinese cancer register covering the past several years [20–29]. A Joinpoint regression model (Joinpoint Regression Program 4.7.0.0) was used to calculate annual growth rates of prevalence and mortality rates (1.5% and 1.9%, respectively) and to predict the corresponding rates in 2020, 2025, and 2030 (assumption: there would be linear increases in the age-specific prevalence and mortality rates from 2015 to 2030). Standard life expectancies (84.2 years) from the GBD 2015 [32] and WHO 2050 (89.4 years) [40] were applied to the model. The standard life expectancies in 2020, 2025, and 2030 (84.9 years, 85.7 years, and 86.4 years, respectively) were estimated linearly. Changes in YLLs, YLDs, and DALYs were predicted for 2020, 2025, and 2030 using a similar approach. The formula was shown in Fig. 1.

### Sensitivity analysis

The sensitivity analysis was based on the single factor analysis with replacing parameters. Limited by the availability of detailed data in China, some parameters were derived from assumptions and foreign studies. A series of sensitivity analyses were carried out for DWs, demographic, and screening parameters associated with breast cancer. Detailed sensitivity analysis factors were shown in Additional files 1 and 2.

## Results

### DALYs in 2015

**Fig. 2:**
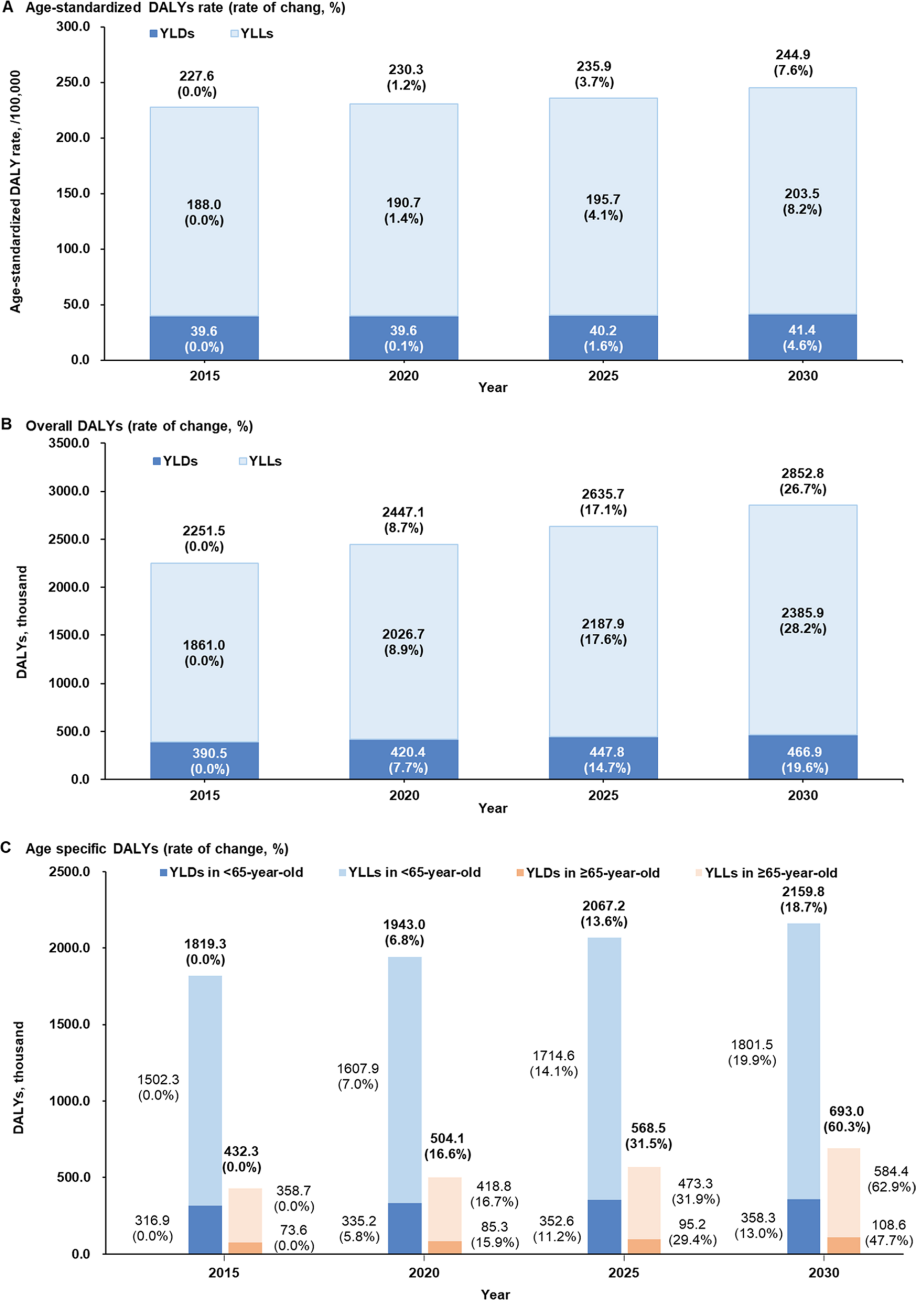
Estimated population-level DALYs attributable to breast cancer in China, 2015–2030. **A** Age-standardized DALY rate (rate of change, compared with the value in 2015, %). **B** Overall number of DALYs (rate of change, %). **C** Age-specific number of DALYs (rate of change, %)

In females in China, 2251.5 thousand breast cancer DALYs were estimated in 2015 (of 82.7% were YLLs: 1861.0 thousand; of 17.3% were YLDs: 390.5 thousand), and the corresponding results reported by the GBD [5] was 2527.0 thousand (of 8.2% were YLDs). The ASDR was 227.6/100,000. Among the overall DALYs, 1819.3 thousand were in breast cancer patients aged < 65 years and 432.3 thousand were in those aged ≥ 65 years, accounting for 80.8% and 19.2% of the total DALYs, respectively. Additional details are provided in Fig. 2.

### DALYs in 2020–2030

If breast cancer screening coverage (25.7%) remains unchanged, the number of breast cancer DALYs in females are expected to increase by 26.7% (60.3% among those aged ≥ 65 years; 18.6% among those aged < 65 years) in 2030 (2852.8 thousand, ASDR: 244.9/100,000). The YLLs (2385.9 thousand, 203.5/100,000) and YLDs (466.9 thousand, 41.4/100,000) would increase by 28.2% and 19.6%, respectively. The DALYs of breast cancer in females aged < 65 years and ≥ 65 years would increase 18.6% and 60.3%, respectively. The DALYs in the ≥ 65 years age group would account for 24.3% of the total DALY burden of breast cancer in 2030. Further details are provided in Fig. 2.

**Fig. 3:**
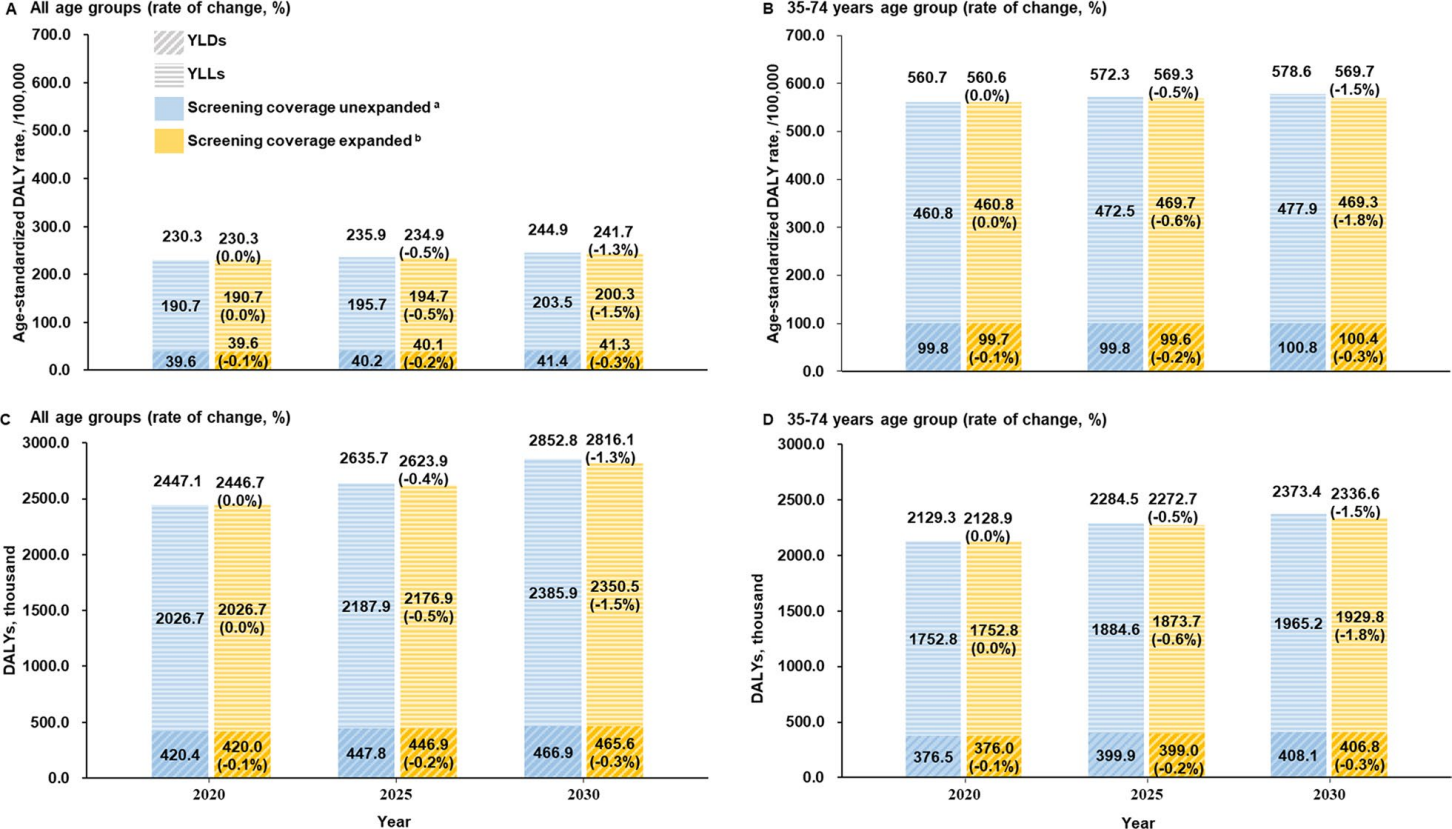
Predicted impact of screening on population-level DALYs attributable to breast cancer in China, 2020–2030. **A** age-standardized DALY rates in all age groups (rate of change, compared with the value in the same year with no expansion of screening coverage, %). **B** Age-standardized DALY rate in the 35–74 years age group (rate of change, %). **C** Overall numbers of DALYs in all age groups (rate of change, %). **D** Number of DALYs in the 35–74 years age group (rate of change, %).^a^Unexpanded screening coverage: always assumed to be 25.7% (individual level) from 2015 to 2030. ^b^Expanded screening coverage: estimated to be 30.7% in 2020, 35.7% in 2025 and 40.7% in 2030 (calculated using data from published literature, deduced from the county-level target in the “Healthy China Initiative, 2019–2030”)

Alternatively, if the individual-level coverage of breast screening expands from 25.7% in 2015 to 40.7% in 2030 (deduced from the “Healthy China Initiative, 2019–2030”), the overall number of breast cancer DALYs (2816.1 thousand) would decrease by 1.3% in the whole female population, compared with the value in the same year without the expansion of coverage; the DALYs would decrease by 1.5% among the screened age group (35–74 years). Additional predicted DALYs and age-standardized results are provided in Fig. 3.

### Sensitivity analysis of DWs

**Fig. 4:**
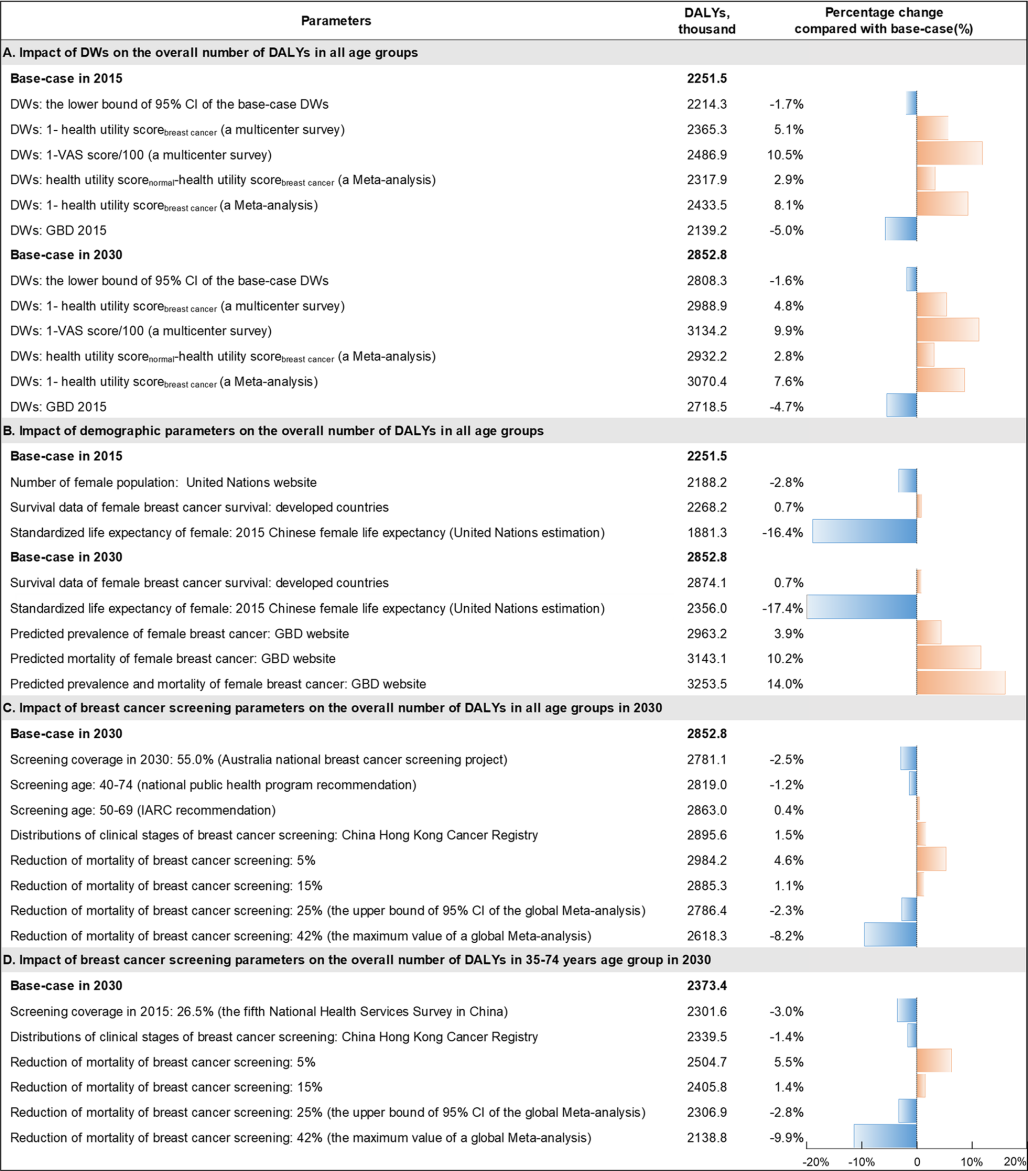
Sensitivity analyses. **A** Impacts of DWs on the overall numbers of DALYs in all age groups. **B** Impacts of demographic parameters on the overall numbers of DALYs in all age groups. **C** Impacts of breast cancer screening-related parameters on the overall numbers of DALYs in all age groups in 2030. **D** Impacts of breast cancer screening-related parameters on the overall number of DALYs in the 35-74 years age group in 2030

The sensitivity analysis found that using local DWs would change ~ 10% of base-case values. The results showed that the approach based on “1-visual analog scale (VAS) score/100” [17–19] had the most significant impact on breast cancer DALYs (increase 10.5%) among the six DWs approaches in 2015. Compared with the base-case DALYs in 2015, the corresponding DALYs using the DWs in the GBD [5] (diagnosis and primary therapy phase: 0.288, controlled phase: 0.049, metastatic phase: 0.451 and terminal phase: 0.569) decreased by 5.0%. The above-mentioned change in the DALYs would be less than 10% in 2030. Further details are provided in Fig. 4A.

### Sensitivity analysis of other key parameters

Sensitivity analyses of demographic and screening parameters showed that: (1) Life expectancy had a strong influence on the DALYs regardless of the years (decreases 16.4% in 2015 and 17.5% in 2030); (2) using predicted prevalence and mortality rates from various databases may change the predicted DALYs in 2030 by 3.9% to 14.0%; and (3) if breast cancer screening coverage expands to 55% in 2030, the DALYs would decrease by 2.5% (among those aged 35–74 years: 3.0%). Further findings regarding impacts from other parameters, including the reduction in cancer mortality due to screening, screening ages, and distributions of cancer clinical stages, are presented in Fig. 4.

## Discussion

Compared with other disease burden indicators, such as incidence and mortality, DALYs are reported in relatively few studies, and even fewer studies have reported DALYs in the Chinese population [7]. This study was the first attempt to use local DWs to calculate the DALYs burden based on the breast cancer prevalence in 2015 among Chinese females, predict the breast cancer DALYs in 2030 under different screening scenarios and estimate the impact of the application of local, detailed DWs. The main findings are as follows: the estimated number of breast cancer DALYs in China was lower overall, but the proportion of YLDs was higher than that reported by GBD, suggesting the importance of applying population-specific DWs and considering other local parameters. If breast cancer screening coverage (25.7%) in China remains unchanged, the breast cancer DALYs would continue to increase (by 26.7%), especially among elderly individuals; alternatively, if the coverage is expanded to 40.7% in 2030, the DALYs would decrease by 1.5% among the screened age groups.

The estimated number of breast cancer DALYs in China was lower overall, but higher in the proportion of YLDs than the number in GBD, suggesting the importance of considering population-specific parameters. (1) A total of 2527.0 thousand breast cancer DALYs in 2015 in China were reported in the GBD [5]. The lower estimate in this study may be explained by the use of local data, including local breast cancer mortality rates, which were lower than those in the GBD [20–29] as well as the Chinese female population [30]. The DALYs estimates were even lower when using the population data reported by the United Nations. (2) YLDs accounted for 17.3% of DALYs, which was higher than that (8.2%) reported in the GBD. This was related to the fact that local DWs values were higher than the DW values in the GBD [5]. Using the DWs for the diagnosis and primary therapy phase, the controlled phase, the metastatic phase and the terminal phase from the GBD [5], the proportion of YLDs decreased to 13.0% in the study, which was close to the reported value in the GBD. Differences were also observed when considering different cancer stage types and the distribution of each stage. The cancer stages in the GBD did not match the typical clinical stages in the Chinese population, so the impact could not be estimated.

This study suggested that if the screening coverage remains unchanged, the DALYs will increase by 26.7% in 2030, especially among the elderly population. The predicted breast cancer incidence and mortality from International Agency for Research on Cancer among Chinese females from 2020 to 2035 (increase 15.0% and 38.1%, respectively) [41] were consistent with those in our study. This study also found that if coverage expands to 40.7% in 2030, the DALYs would decrease by 1.5% among females aged 35–74 years, which was less than expected. One possible explanation is that breast cancer screening slightly impacts the reduction in mortality for a short duration (approximately 20% for ten years), and this study was limited by the approximately ten-year observation. In other words, even with ideally complete coverage in 2020, there would be an approximately 20% reduction in mortality by 2030. However, the breast cancer screening coverage in China is still gradually expanding under government promotion. Considering the number of the Chinese population, it cannot achieve full coverage in the near future. Therefore, the population-based breast cancer screening and other secondary prevention strategies need long-term and robust promotion.

Mainly enabled by detailed DWs associated with cancer patients in China, along with some integrated key demographic parameters and breast cancer and screening parameters, as well as the governmental goals for cancer control, the current prevalence-based analysis is, to the best of our knowledge, the most detailed exploratory DALY estimation focusing on breast cancer, and this approach could potentially be extended to broader cancer types and diseases. The findings also suggest the importance of applying the population- and cancer-specific DWs in estimating the DALYs. DWs should be given more attention in future related research and evaluations, in both China and worldwide.

The current study has several limitations. (1) Methodology: One of the purposes of our study was to provide a relatively simple method for calculating DALYs based on locally available parameters. Although the simple DALY model based on prevalence was relatively feasible, more accurate estimations could not be achieved. For example, precancerous lesions and their influence on the incidence were not considered, and the prognoses of different clinical stages were not distinguished. Additionally, the health utility values associated with these disease statuses have not been reported. (2) Data of local parameters were unavailable: Some data on factors such as breast cancer prevalence and survival rates, screening coverage and outcomes were hypothesized or indirectly assumed in this study; this may increase the instability of the results, although sensitivity analyses were performed. In future studies, additional available parameters will be summarized to support our calculation. (3) Short prediction duration: a larger reduction in the DALYs is expected to be observed when more robust local data are available in the future, with long-term projections. In the future study, we will use complex models to calculate DALYs more accurately.

## Conclusions

The global platform (GBD) data are a convenient and comparable resource for cancer prevention and control analyses worldwide. Population-level and population-specific DALYs are of great necessity. Due to promotion by the government and the development of academic research, several large-scale screening intervention projects for secondary cancer prevention have been gradually initiated. The localized disease burden estimate is particularly helpful and meaningful to the evaluation of innovative interventions and the development of prompt and accurate guidance for decision-makers. Future research directions should be considered: (1) according to the definitions of cancer stages in the GBD [5], and matching and quantifying local DWs in China is a promising approach; (2) further studies could optimize the DALYs estimation based on prevalence, and carry out more accurate calculations using life tables, Markov multi-queue models and other models.

## Supplementary Information

**Additional file 1**: Demographic, breast cancer epidemiological and screening factors of the female populations in China.

**Additional file 2**: Clinical stage-specific disability weights and distribution of breast cancer in females in China.

**Additional file 3**: Age-specific incidence, mortality and survival rates of breast cancer.

**Additional file 4**: Age-specific number of females and standardized life expectancy

## Data Availability

Data are publicly available.

## Abbreviations

DALYs: Disability-adjusted life years
YLDs: Years lived with disability
YLLs: Years of life lost
DWs: Disability weights
GBD: Global burden of disease study
95% CI: 95% confidence interval
