# Supplementary material for: Estimating disability-adjusted life years for breast cancer and the impact of screening in female populations in China, 2015–2030: an exploratory prevalence-based analysis applying local weights
Source: Popul Health Metr. 2022 Oct 7;20:19. doi: 10.1186/s12963-022-00296-1 (PMC9547451; doi:10.1186/s12963-022-00296-1)
Supplement: Supplementary file 1 — Additional file 1: Demographic, breast cancer epidemiological and screening factors of the female populations in China. [file 12963_2022_296_MOESM1_ESM.docx]

Additional file 1. Demographic, Breast Cancer Epidemiological and Screening Factors of the Female Population in China

| Parameters | | | Values | Explanation for parameters selection | Reference |
| --- | --- | --- | --- | --- | --- |
| Demographic parameters | | | |  |  |
|  | Female population in China, 10^9^ | | |  |  |
|  |  | Base-case analysis | 2015: 6.7  2030: 7.2 | 2006-2015 data from China Statistical Yearbooks  2020-2030 data from the United Nations website, detailed year- and age-specific data were shown in Additional file 3 | [1, 2] |
|  |  | Sensitivity analysis | 2015: 6.8 | Data from the United Nations website | [2] |
|  | Female standardised life expectancy in 2015, year | | | |  |
|  |  | Base-case analysis | 84.2 | Data from the standard life expectancy estimated by GBD 2015, used as the base-case parameter for external comparison, detailed year- and age-specific life expectancy data were shown in Additional file 3 | [3] |
|  |  | Sensitivity analysis | 77.5 | Data from China’s females estimated by the United Nations in 2015, detailed data shown in Additional file 3 | [2] |
|  | Female standardised life expectancy in 2030, year | | | |  |
|  |  | Base-case analysis | 86.4 | According to the standard life expectancy in 2015 reported by GBD 2015 and in 2050 reported by WHO, the standard life expectancy in 2030 was estimated linearly, detailed age-specific data were shown in Suppl. Table 3 | [3, 4] |
|  |  | Sensitivity analysis | 80.5 | Data from China’s females estimated by the United Nations in 2030, detailed data shown in Additional file 3 | [2] |
| Breast cancer epidemiological parameters | | | | |  |
|  | Incidence rate, /100,000 | | 2006: 29.3  2015: 26.3 | The incidence rates from 2006 to 2015 were used to estimate the prevalence cases in 2015, detailed year- and age-specific data were shown in Additional file 4 | [5-14] |
|  | Mortality rate, /100,000 | | 2015: 6.3 | The mortality rates from 2006 to 2015 were used to estimate the death cases in 2015, detailed year- and age-specific data were shown in Additional file 4 | [14] |
|  | Survival rate, % | |  |  |  |
|  |  | Base-case analysis | 82.0 | Data from 2012-2015 Chinese local data, detailed year and age specific data were shown in Additional file 2 | [15] |
|  |  | Sensitivity analysis | 89.0 | Data from the developed countries | [16] |
| Breast cancer screening parameters | | | | |  |
|  | Screening coverage, % | | | |  |
|  |  | Base-case analysis for 2015 | 2015: 25.7 | Data from a Chinese population-level study in 2015 | [17] |
|  |  | Base-case analysis for 2020-2030 | 2020: 30.7  2025: 35.7  2030: 40.7 | A Chinese population-level study reported the screening coverage rate for breast cancer was 25.7% in 2015, and increased approximately 1% per year from 2013 to 2015. Accordingly, the coverage rates (at the individual level) of breast cancer screening in Chinese females were estimated to be 30.7%, 35.7%, and 40.7% for 2020, 2025, and 2030, respectively. | [17, 18] |
|  |  | Sensitivity analysis for 2015 | 2015: 26.5 | Data from the fifth National Health Services Survey in China. | [19] |
|  |  | Sensitivity analysis for 2030 | 2030: 55.0 | Data from Australia national breast cancer screening project started in 1991, which was one of the highest screening coverage rate of breast cancer in the world | [20] |
|  | Ten-year reduction of breast cancer mortality after screening, % | | | |  |
|  |  | Base-case analysis | 22.0 | Data from a latest global Meta-analysis (including 27 studies) reported that the integrated mortality reduction rate was 22% (95% CI: 18% - 25%) | [21] |
|  |  | Sensitivity analysis | 5.0-42.0 | According to the results of a global Meta-analysis and hypothesis: ‘42%’ from the maximum value of the Meta-analysis; since there were differences among the included studies, the sensitivity analyses tried the values of 25% (upper 95% CI of the Meta-analysis results), 15%, and 5% in turn | [21] |

*GBD* the Global Burden of Disease Study, *95% CI* 95% confidence interval

**REFERENCE**

1. National Bureau of Statistics. China Statistical Yearbooks. Available at: http://www.stats.gov.cn/tjsj/ndsj/. Accessed January 12, 2021.

2. United Nations: Department of Economic and Social Affairs Population Dynamics. World Population Prospects 2019. Available at: https://population.un.org/wpp/DataQuery/. Accessed January 12, 2021.

3. Institute for Health Metrics and Evaluation (IHME). Global Burden of Disease Study 2015 (GBD 2015) Reference Life Table. Available at: http://ghdx.healthdata.org/record/ihme-data/gbd-2015-reference-life-table. Accessed January 12, 2021.

4. World Health Organization. WHO methods and data sources for global burden of disease estimates 2000-2019. Available at: www.who.int/data/gho/data/themes/mortality-and-global-health-estimates/global-health-estimates-leading-causes-of-dalys. Accessed January 12, 2021.

5. Zhao P, Chen WQ. 2009 Chinese cancer registry annual report. Military Medical Science Press; 2010.

6. Zhao P, Chen WQ. 2010 Chinese cancer registry annual report. Military Medical Science Press; 2011.

7. He J, Zhao P, Chen WQ. 2011 Chinese cancer registry annual report. Military Medical Science Press; 2012.

8. He J, Chen WQ. 2012 Chinese cancer registry annual report. Military Medical Science Press; 2012.

9. He J, Chen WQ. 2013 Chinese cancer registry annual report. Tsinghua University Press; 2017.

10. He J, Chen WQ. 2014 Chinese cancer registry annual report. Tsinghua University Press; 2017.

11. He J, Chen WQ. 2015 Chinese cancer registry annual report. Tsinghua University Press; 2017.

12. He J, Chen WQ. 2016 Chinese cancer registry annual report. Tsinghua University Press; 2017.

13. He J, Chen WQ. 2017 Chinese cancer registry annual report. People’s Medical Publishing House; 2018

14. He J, Chen WQ. 2018 Chinese cancer registry annual report. People’s Medical Publishing House; 2019.

15. Zeng H, Chen W, Zheng R, et al. Changing cancer survival in China during 2003–15: a pooled analysis of 17 population-based cancer registries. Lancet Glob Health. 2018;6(5):e555-e567.

16. Surveillance, Epidemiology, and End Results (SEER) Program (www.seer.cancer.gov) SEER*Stat Database: Incidence - SEER Research Data, 13 Registries, Nov 2019 Sub (1992-2017) - Linked To County Attributes - Time Dependent (1990-2017) Income/Rurality, 1969-2018 Counties, National Cancer Institute, DCCPS, Surveillance Research Program, released April 2020, based on the November 2019 submission.

17. Zhang M, Zhong Y, Bao H, , et al: Breast Cancer Screening Rates Among Women Aged 20 Years and Above - China, 2015. China CDC Wkly 2021, 3:267-273.

18. Bao HL, Wang LH, Wang LM, et al. Study on the coverage of cervical and breast cancer screening among women aged 35-69 years and related impact of socioeconomic factors in China, 2013. Zhonghua Liu Xing Bing Xue Za Zhi 2018, 39:208-212.

19. Center for Health Statistics and Information. National Health and Family Planning Commission. An Analysis Report of National Health Services Survey in China. Available at: http://www.nhc.gov.cn/ewebeditor/uploadfile/2016/10/20161026163512679.pdf. Accessed January 12, 2021.

20 Lew J B, Feletto E, Wade S, et al. Benefits, harms and cost-effectiveness of cancer screening in Australia: an overview of modelling estimates. Public Health Res Pract. 2019;29(2):e29121913.

21. Dibden A, Offman J, Duffy SW, et al. Worldwide review and meta-analysis of cohort studies measuring the effect of mammography screening programmes on incidence-based breast cancer mortality. Cancers (Basel). 2020;12(4):976.
