# Supplementary material for: Estimating disability-adjusted life years for breast cancer and the impact of screening in female populations in China, 2015–2030: an exploratory prevalence-based analysis applying local weights
Source: Popul Health Metr. 2022 Oct 7;20:19. doi: 10.1186/s12963-022-00296-1 (PMC9547451; doi:10.1186/s12963-022-00296-1)
Supplement: Supplementary file 2 — Additional file 2: Clinical stage-specific disability weights and distribution of breast cancer in females in China. [file 12963_2022_296_MOESM2_ESM.docx]

Additional file 2. Clinical Stage-specific Disability Weights and Distribution of Breast Cancer in Females in China

| Parameters | | Clinical stages of breast cancer | | | | Explanation for parameters selection | Reference |
| --- | --- | --- | --- | --- | --- | --- | --- |
|  |  | I | II | III | IV |  |  |
| Disability weights^a^ | |  |  |  |  |  |  |
|  | Base-case analysis | 0.162 | 0.158 | 0.176 | 0.265 | Data from a multicenter hospital-based survey, with 2626 breast cancer patients from 12 provinces in China;  DWs approach: health utility score_normal_ - health utility score_breast cancer_ | [1-3] |
|  | Sensitivity analysis 1 | 0.142 | 0.146 | 0.158 | 0.227 | Data from a multicenter survey;  DWs approach: the lower bound of 95%CI of DWs above study |  |
|  | Sensitivity analysis 2 | 0.210 | 0.206 | 0.225 | 0.314 | Data from a multicenter survey;  DWs approach: 1 - health utility score_breast cancer_ | [1,2] |
|  | Sensitivity analysis 3 | 0.264 | 0.258 | 0.275 | 0.347 | Data from a multicenter survey;  DWs approach: 1 - Visual Analogue Scale (VAS) score / 100 |  |
|  | Sensitivity analysis 4 | 0.181 | 0.191 | 0.211 | 0.221 | Data from a Meta-analysis;  DWs approach: health utility score_normal_ - health utility score_breast cancer_ | [2,3] |
|  | Sensitivity analysis 5 | 0.230 | 0.240 | 0.260 | 0.270 | Data from a Meta-analysis;  DWs approach: 1 - health utility score_breast cancer_ | [2] |
| Distribution of clinical stages, % | | | | | | |  |
|  | Base-case analysis: in populations without screening | 19.2 | 54.7 | 22.8 | 3.3 | Data from a multicenter hospital-based clinical epidemiologic survey, with 4211 breast cancer patients from 7 provinces in China | [5] |
|  | Base-case analysis: in populations with screening | 35.9 | 49.0 | 14.4 | 0.7 | Data from a ‘central transfer payments for breast cancer screening program’, with 0.4 million women from 30 provinces in China | [6] |
|  | Sensitivity analysis: in populations with screening | 52.1 | 38.2 | 5.7 | 4.1 | Data from China Hong Kong Cancer Registry | [7] |

*DWs* disability weights, *YLDs* years lived with disability, *GBD* the Global Burden of Disease Study

^a^ In sensitivity analysis, different-category DWs for generic cancers (not BC specific) from the GBD were also tried, which were 0.288 for the diagnosis and primary therapy phase, 0.049 for the controlled phase, 0.451 for the metastatic phase, and 0.569 for the terminal phase.

**REFERENCE**

1. Wang L, Shi J F, Zhu J, et al. Health-related quality of life and utility scores of patients with breast neoplasms in China: A multicenter cross-sectional survey. *Breast.* 2018;39:53-62. https://doi.org/10.1016/j.breast.2018.03.004
2. Zhu J, Yan XX, Dai M, Chen WQ, Shi JF. Approaches to derive disability weights based on EQ-5D measurement: a systematic review. *Chin J Evid-Based Med*. 2020;20:782-788.
3. Guan HJ, Liu GE. Comparison Analysis on Health Related Quality of Life among Urban and Rural Residents in 4 Cities of China. *Chin Health Econ*. 2015;34:5-12.
4. Zhu J, Wang L, He SJ, et al. Health utility score of breast cancer in China: a systematic review. *Chin J Evid-Based Med*. 2017;17(9):1066-1071.
5. Wang Q, Li J, Zheng S, et al. Breast cancer stage at diagnosis and area-based socioeconomic status: a multicenter 10-year retrospective clinical epidemiological study in China. *BMC cancer*. 2012;12:122. https://doi.org/10.1186/1471-2407-12-122
6. Gao Y. Development and Health Economic Evaluation of Breast Cancer Screening Strategy Among Chinese Women. [master's thesis]. Tianjin: Tianjin Medical University; 2016.
7. Wong IOL, Kuntz KM, Cowling BJ, et al. Cost effectiveness of mammography screening for Chinese women. Cancer. 2007; 110(4):885-95. <https://doi.org/10.1002/cncr.22848>
