# Supplementary material for: Estimating disability-adjusted life years for breast cancer and the impact of screening in female populations in China, 2015–2030: an exploratory prevalence-based analysis applying local weights
Source: Popul Health Metr. 2022 Oct 7;20:19. doi: 10.1186/s12963-022-00296-1 (PMC9547451; doi:10.1186/s12963-022-00296-1)
Supplement: Supplementary file 3 — Additional file 3: Age-specific incidence, mortality and survival rates of breast cancer. [file 12963_2022_296_MOESM3_ESM.docx]

Additional file 3. Age-specific Incidence, Mortality and Survival Rates of Breast Cancer

| **Age**  **group ^a^**  **, years** | **Incidence rate ^b^, /100 000** | | | | | | | | | | **Mortality**  **rate, 2015 ^b^,**  **/100 000** | **5-year**  **Survival**  **rate ^c^, %** |
| --- | --- | --- | --- | --- | --- | --- | --- | --- | --- | --- | --- | --- |
|  | **2006** | **2007** | **2008** | **2009** | **2010** | **2011** | **2012** | **2013** | **2014** | **2015** |  |  |
| 15-19 | 0.2 | 0.1 | 0.2 | 0.2 | 0.1 | 0.0 | 0.2 | 0.3 | 0.2 | 0.2 | 0.0 | 74.1 |
| 20-24 | 0.7 | 0.6 | 1.1 | 1.2 | 1.5 | 1.1 | 1.3 | 1.2 | 1.7 | 1.4 | 0.2 | 76.3 |
| 25-29 | 4.2 | 4.5 | 4.9 | 4.5 | 4.9 | 4.8 | 5.2 | 5.0 | 5.4 | 6.0 | 0.7 | 77.8 |
| 30-34 | 13.4 | 12.5 | 14.5 | 13.6 | 14.2 | 14.2 | 13.0 | 15.3 | 15.1 | 15.7 | 1.9 | 80.1 |
| 35-39 | 27.7 | 31.1 | 33.2 | 30.0 | 31.0 | 34.8 | 30.3 | 30.2 | 30.7 | 30.4 | 4.3 | 82.9 |
| 40-44 | 60.3 | 61.7 | 61.3 | 52.9 | 54.8 | 63.5 | 58.6 | 59.1 | 57.4 | 59.5 | 8.6 | 86.4 |
| 45-49 | 88.8 | 86.2 | 92.1 | 83.9 | 80.6 | 95.5 | 86.1 | 80.8 | 78.2 | 80.3 | 12.2 | 87.0 |
| 50-54 | 97.5 | 104.4 | 108.3 | 100.8 | 90.2 | 104.1 | 84.7 | 85.7 | 89.2 | 90.1 | 17.2 | 87.0 |
| 55-59 | 96.3 | 98.3 | 103.4 | 95.8 | 93.8 | 111.2 | 95.9 | 93.9 | 92.0 | 88.0 | 20.8 | 84.5 |
| 60-64 | 80.9 | 82.1 | 89.0 | 88.7 | 85.2 | 99.6 | 87.1 | 87.9 | 91.8 | 92.4 | 24.7 | 84.5 |
| 65-69 | 80.0 | 79.8 | 89.2 | 77.8 | 72.0 | 94.9 | 78.4 | 75.5 | 76.2 | 79.9 | 22.9 | 84.3 |
| 70-74 | 85.6 | 85.3 | 90.2 | 80.0 | 70.6 | 91.9 | 72.2 | 70.3 | 70.4 | 66.4 | 24.3 | 84.3 |
| 75-79 | 71.1 | 81.3 | 81.4 | 74.3 | 69.3 | 88.0 | 66.4 | 62.9 | 63.3 | 61.1 | 30.7 | 83.0 |
| 80-84 | 67.0 | 65.9 | 59.1 | 63.3 | 61.7 | 78.0 | 52.7 | 54.1 | 54.8 | 51.1 | 41.4 | 80.9 |
| 85+ | 49.6 | 50.4 | 55.8 | 50.6 | 53.6 | 52.2 | 43.5 | 37.6 | 36.5 | 39.4 | 48.4 | 75.2 |
| Crude rate | 42.0 | 43.2 | 47.6 | 42.6 | 40.7 | 50.0 | 41.8 | 41.7 | 42.1 | 42.6 | 10.2 | - |
| ASR | 29.3 | 29.9 | 31.7 | 29.0 | 27.9 | 32.8 | 28.3 | 28.0 | 28.2 | 28.3 | 6.9 | - |

*ASR* age-standardized rate

^a^ In the calculation, the incidence rate and mortality rate of the 0-14 years group were considered as 0 and survival rate as 1.

^b^ Data source: the incidence and mortality rates were extracted from Chinese cancer registration annual reports.^1-10^

^c^ Base-case analysis data source: According to the 5-year survival rate of breast cancer in 2012-2015 in China reported by Zeng et al.,^11^ the estimated values were read by Engauge Digitizer 11.1.^12^ However, the study results reported five broad age groups (≤45y, 45-54y, 55-64y, 65-74y and ≥75y).Considering no more detailed local data supporting, we assumed that the values of 45-49y and 50-54y were assumed as the value of 45-54y (55-64y and 65-74y groups were the same process).And based on the survival rate ratios between age groups using SEER 5-year survival rates,^13^ we extrapolated the age-specific survival rates not reported in the local study. Then using the survival ratios reported by SEER in adjacent years,^13^ we derived the 1- to 9-year survival rates for breast cancer in China. The sensitivity analysis data were extracted from the SEER database,^13^ and the corresponding values of 15 age groups from 15-19 to 85+ were 73.7%, 76.3%, 77.3%, 80.3%, 83.4%, 87.6%, 88.8%, 88.2%, 88.5%, 89.2%, 89.3%, 88.6%, 87.5%, 85.1% and 79.4%, respectively.

**REFERENCE**

1. Zhao P, Chen WQ. 2010 Chinese cancer registry annual report. Military Medical Science Press; 2011.
2. He J, Zhao P, Chen WQ. 2011 Chinese cancer registry annual report. Military Medical Science Press; 2012.
3. He J, Chen WQ. 2012 Chinese cancer registry annual report. Military Medical Science Press; 2012.
4. He J, Chen WQ. 2013 Chinese cancer registry annual report. Tsinghua University Press; 2017.
5. He J, Chen WQ. 2014 Chinese cancer registry annual report. Tsinghua University Press; 2017.
6. He J, Chen WQ. 2015 Chinese cancer registry annual report. Tsinghua University Press; 2017.
7. He J, Chen WQ. 2016 Chinese cancer registry annual report. Tsinghua University Press; 2017.
8. He J, Chen WQ. 2017 Chinese cancer registry annual report. People’s Medical Publishing House; 2018
9. He J, Chen WQ. 2018 Chinese cancer registry annual report. People’s Medical Publishing House; 2019.
10. Zeng H, Chen W, Zheng R, et al. Changing cancer survival in China during 2003–15: a pooled analysis of 17 population-based cancer registries. *Lancet Glob Health*. 2018;6(5):e555-e567.
11. National Health Commission of the People’s Republic of China. Reply to proposal No. 2364 of the first session of the 13th National People's Congress. Available at: http://www.nhc.gov.cn/wjw/jiany/201812/f4411e8294604f79a511cbadb262f6a2.shtml. Accessed January 12, 2021.
12. Mitchell M, Muftakhidinov B, Winchen T, et al, Engauge Digitizer Software. Accessed January 12, 2021. http://markummitchell.github.io/engauge-digitizer.
13. Surveillance, Epidemiology, and End Results (SEER) Program (www.seer.cancer.gov) SEER*Stat Database: Incidence - SEER Research Data, 13 Registries, Nov 2019 Sub (1992-2017) - Linked To County Attributes - Time Dependent (1990-2017) Income/Rurality, 1969-2018 Counties, National Cancer Institute, DCCPS, Surveillance Research Program, released April 2020, based on the November 2019 submission.
