# Supplementary material for: Estimating disability-adjusted life years for breast cancer and the impact of screening in female populations in China, 2015–2030: an exploratory prevalence-based analysis applying local weights
Source: Popul Health Metr. 2022 Oct 7;20:19. doi: 10.1186/s12963-022-00296-1 (PMC9547451; doi:10.1186/s12963-022-00296-1)
Supplement: Supplementary file 4 — Additional file 4: Age-specific number of females and standardized life expectancy [file 12963_2022_296_MOESM4_ESM.docx]

Additional file 4. Age-specific Number of Females and Standardized Life Expectancy

| **Age group**  **, years** | **Number of females ^a^** | | | | | | | | | |  | **Number of projected females ^b^** | | |  | **Standardized life expectancy, years** | | | |
| --- | --- | --- | --- | --- | --- | --- | --- | --- | --- | --- | --- | --- | --- | --- | --- | --- | --- | --- | --- |
|  | **2006** | **2007** | **2008** | **2009** | **2010** | **2011** | **2012** | **2013** | **2014** | **2015** |  | **2020** | **2025** | **2030** |  | **2015**  **base-case analysis ^c^** | **2015 sensitivity analysis ^d^** | **2030**  **base-case analysis ^e^** | **2030**  **sensitivity analysis ^d^** |
| 0-4 | 30 249 173 | 29 814 444 | 30 503 946 | 30 948 454 | 34 470 044 | 34 803 529 | 35 243 081 | 35 545 012 | 35 889 464 | 37 094 510 |  | 39 476 105 | 36 234 058 | 34 026 171 |  | 84.2 | 76.9 | 86.4 | 78.9 |
| 5-9 | 34 890 849 | 34 363 333 | 32 793 687 | 32 412 371 | 32 416 884 | 32 984 706 | 33 762 936 | 34 739 659 | 35 039 239 | 34 583 755 |  | 40 415 039 | 39 398 851 | 36 167 828 |  | 79.3 | 72.3 | 81.6 | 74.2 |
| 10-14 | 45 291 069 | 43 262 222 | 41 394 589 | 38 499 427 | 34 641 185 | 33 849 412 | 33 062 575 | 32 143 552 | 32 441 127 | 32 561 451 |  | 38 912 828 | 40 353 824 | 39 344 410 |  | 74.3 | 67.4 | 76.6 | 69.3 |
| 15-19 | 54 621 830 | 51 645 556 | 48 848 929 | 45 068 729 | 47 984 284 | 45 084 706 | 42 123 947 | 39 583 942 | 36 655 761 | 34 784 283 |  | 38 238 737 | 38 775 600 | 40 224 163 |  | 69.4 | 62.4 | 71.6 | 64.4 |
| 20-24 | 42 876 516 | 44 534 444 | 46 091 319 | 49 983 963 | 63 403 945 | 62 732 941 | 59 818 291 | 56 503 650 | 53 390 230 | 48 096 568 |  | 40 884 302 | 38 000 411 | 38 550 873 |  | 64.4 | 57.5 | 66.6 | 59.4 |
| 25-29 | 42 163 175 | 43 900 000 | 43 878 241 | 43 302 405 | 50 176 814 | 52 096 471 | 53 765 343 | 56 500 000 | 59 654 405 | 63 538 548 |  | 46 466 160 | 40 628 565 | 37 764 689 |  | 59.5 | 52.7 | 61.7 | 54.5 |
| 30-34 | 52 458 655 | 49 033 333 | 46 674 183 | 45 705 613 | 47 616 381 | 47 341 176 | 49 394 705 | 49 502 433 | 49 587 937 | 50 242 130 |  | 62 295 742 | 46 214 741 | 40 406 105 |  | 54.6 | 47.8 | 56.7 | 49.6 |
| 35-39 | 63 858 875 | 64 744 444 | 62 310 034 | 61 238 259 | 57 634 855 | 55 676 471 | 52 382 671 | 50 215 328 | 48 692 707 | 47 635 013 |  | 48 745 948 | 62 004 795 | 45 990 793 |  | 49.7 | 43.0 | 51.8 | 44.8 |
| 40-44 | 64 503 859 | 64 664 444 | 64 342 728 | 64 712 486 | 61 145 286 | 61 864 706 | 63 321 300 | 61 743 309 | 60 662 754 | 57 582 123 |  | 46 984 787 | 48 468 164 | 61 689 278 |  | 44.8 | 38.1 | 46.9 | 39.9 |
| 45-49 | 42 168 688 | 42 377 778 | 48 072 153 | 53 515 464 | 51 818 135 | 58 140 000 | 58 661 853 | 58 692 214 | 59 351 535 | 60 956 070 |  | 58 664 268 | 46 641 663 | 48 137 376 |  | 40.0 | 33.4 | 42.0 | 35.1 |
| 50-54 | 49 574 421 | 50 953 333 | 51 485 908 | 47 987 400 | 38 389 937 | 35 744 706 | 36 536 703 | 41 579 075 | 46 755 070 | 51 433 685 |  | 61 097 362 | 58 059 849 | 46 186 514 |  | 35.2 | 28.7 | 37.2 | 30.4 |
| 55-59 | 37 196 251 | 40 270 000 | 43 134 160 | 45 271 478 | 40 229 536 | 41 709 412 | 42 439 230 | 42 368 613 | 39 684 459 | 37 831 477 |  | 48 782 446 | 60 087 817 | 57 164 392 |  | 30.6 | 24.2 | 32.5 | 25.8 |
| 60-64 | 26 393 605 | 27 833 333 | 29 232 244 | 31 319 588 | 28 832 856 | 30 611 765 | 33 091 456 | 35 311 436 | 37 495 039 | 39 119 330 |  | 38 596 854 | 47 353 201 | 58 458 487 |  | 26.0 | 19.9 | 27.8 | 21.4 |
| 65-69 | 21 539 140 | 21 726 667 | 21 827 508 | 22 722 795 | 20 364 811 | 21 164 706 | 22 684 717 | 23 890 511 | 25 709 876 | 27 438 355 |  | 37 622 978 | 36 541 479 | 45 022 538 |  | 21.6 | 15.9 | 23.3 | 17.3 |
| 70-74 | 18 246 968 | 18 723 333 | 18 879 369 | 18 595 647 | 16 568 944 | 16 978 824 | 17 128 761 | 17 222 628 | 17 765 929 | 18 496 075 |  | 23 524 526 | 34 020 143 | 33 297 383 |  | 17.3 | 12.4 | 18.8 | 13.6 |
| 75-79 | 11 886 439 | 12 127 778 | 12 903 044 | 13 208 477 | 11 278 859 | 13 195 294 | 13 388 688 | 13 624 088 | 13 563 459 | 13 908 773 |  | 14 337 340 | 19 679 877 | 28 831 177 |  | 13.4 | 9.5 | 14.7 | 10.4 |
| 80-84 | 6 858 875 | 7 057 778 | 7 316 798 | 7 578 465 | 7 455 696 | 7 811 765 | 8 085 439 | 8 529 197 | 8 881 748 | 9 128 811 |  | 9 297 788 | 10 582 594 | 14 812 864 |  | 9.8 | 7.1 | 11.0 | 7.8 |
| 85+ | 3 912 900 | 4 081 111 | 4 406 990 | 4 416 953 | 4 758 898 | 4 880 000 | 4 925 391 | 5 330 900 | 5 809 262 | 6 049 497 |  | 6 733 224 | 8 402 052 | 10 106 439 |  | 6.8 | 5.3 | 7.2 | 5.8 |

^a^ Data source: the actual population data extracted from the China Statistical Yearbook 2007-2016.^1^

^b^ Data source: the projected population extracted from the United Nations World Population Projections.^2^

^c^ Data source: the standard Life expectancy reported by GBD 2015.^3^

^d^ Data source: the life expectancy of Chinese females reported by United Nations World Population Projections.^3^

^e^ Data source: according to the standard life expectancy in 2015 reported by GBD 2015 and in 2050 reported by WHO, the standard life expectancy in 2030 was estimated linearly.

**REFERENCE**

1. National Bureau of Statistics. China Statistical Yearbooks. Available at: http://www.stats.gov.cn/tjsj/ndsj/. Accessed January 12, 2021.
2. United Nations: Department of Economic and Social Affairs Population Dynamics. World Population Prospects 2019. Available at: https://population.un.org/wpp/DataQuery/. Accessed January 12, 2021.
3. Institute for Health Metrics and Evaluation (IHME). Global Burden of Disease Study 2015 (GBD 2015) Reference Life Table. Available at: http://ghdx.healthdata.org/record/ihme-data/gbd-2015-reference-life-table. Accessed January 12, 2021.
